# Combined Awake Craniotomy with Endoscopic Port Surgery for Resection of a Deep-Seated Temporal Lobe Glioma: A Case Report

**DOI:** 10.1155/2013/401359

**Published:** 2013-04-29

**Authors:** Lance Bodily, Arlan H. Mintz, Johnathan Engh

**Affiliations:** ^1^Department of Neurological Surgery, University of Pittsburgh School of Medicine, UPMC Presbyterian, Suite B-400, 200 Lothrop Street, Pittsburgh, PA 15213, USA; ^2^Capital Insititute for Neurosciences, Two Capital Way, Suite 456, Pennington, NJ 08534, USA

## Abstract

The authors describe the combination of awake craniotomy and minimally invasive endoscopic port surgery to resect a high-grade glioma located near eloquent structures of the temporal lobe. Combined minimally invasive techniques such as these may facilitate deep tumor resection within eloquent regions of the brain, allowing minimum white matter dissection. Technical aspects of this procedure, a case outcome involving this technique, and the direction of further investigations for the utility of these techniques are discussed.

## 1. Introduction

Awake craniotomy with cortical mapping has served as the gold standard for functional preservation in brain tumor surgery since its introduction over half a century ago [1]. For the resection of tumors near the motor cortex or language pathway areas, this technique can help maximize the volume of tumor removal, while minimizing the risk of iatrogenic injury [2–4]. 

For deep-seated brain tumors, resection using a minimally invasive cylindrical retractor has the potential to minimize damage to the surrounding white matter incurred as a result of the surgical channel into the tumor. This approach, initially championed by Kelly [5–7], has been modified into an even less invasive endoscopic port surgery in more recent years. Endoscopic port surgery (EPS) has been described for the removal of both intraventricular and intraparenchymal tumors [8–11]. 

In this paper, the authors describe the combination of awake craniotomy with electrophysiological brain mapping and EPS to resect a glioblastoma of the dominant temporal lobe. The case is an example of a deep-seated, eloquent tumor for which the combination of the two techniques seemed intuitive in order to facilitate a favorable outcome. 

## 2. Case Report

### 2.1. Patient Presentation

A 63-year-old, right-handed man presented with a 6-month history of gait deterioration, requiring a walker. For the 3-4 weeks prior to presentation, he had also experienced a new onset of memory difficulties and subtle trouble with word finding, as well as mild headaches and declining vision. Of note, the patient had a history of spinal cerebellar atrophy (SCA). The patient's neurologic exam was remarkable for mild dysmetria, dysdiadochokinesia, and a positive Romberg sign, in addition to subtle fluent dysphasia and memory dysfunction. 

**Figure 1:**
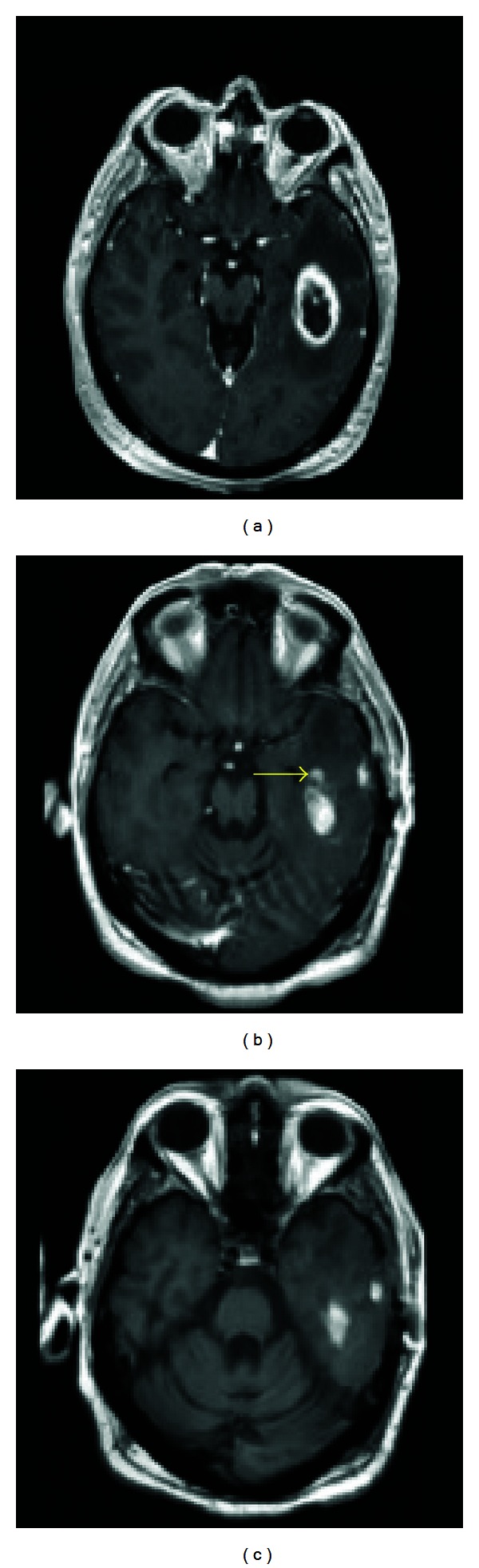
Preoperative and postoperative images. (a) Preresection T1 axial MRI with contrast showing a temporal lesion with an estimated 5.0 cubic centimeter volume. (b) Immediate postresection T1 axial MRI with contrast showing a slight amount of residual enhancement at the anteromedial margin of the resection cavity. (Yellow arrow indicates residual tumor area not present in (c)). (c) The corresponding noncontrast enhanced T1 MRI image taken at the same time demonstrates that the posteromedial and lateral hyperintensities are in fact blood products and postoperative change rather than gross residual tumor. A 93.3% volumetric resection was achieved.

An MRI scan revealed a left temporal lobe mass measuring 3.5 cm × 2 cm abutting the temporal horn of the lateral ventricle (Figure 1). Notable T2 signal prolongation was observed, extending from the ventricle to more anterior and superior portions of the temporal lobe. Mass effect was present, with mild effacement of the ventricle and minimal midline shift. Radiological signs secondary to the SCA were also observed, including cerebellar atrophy with enlargement of the foramina of Luschka bilaterally, as well as a fourth ventricular enlargement. 

A craniotomy for tumor resection was recommended to the patient in order to achieve a tissue diagnosis, alleviate neurologic symptoms, and extend survival given the presumptive diagnosis of high grade glioma.

### 2.2. Operation and Operative Course

**Figure 2:**
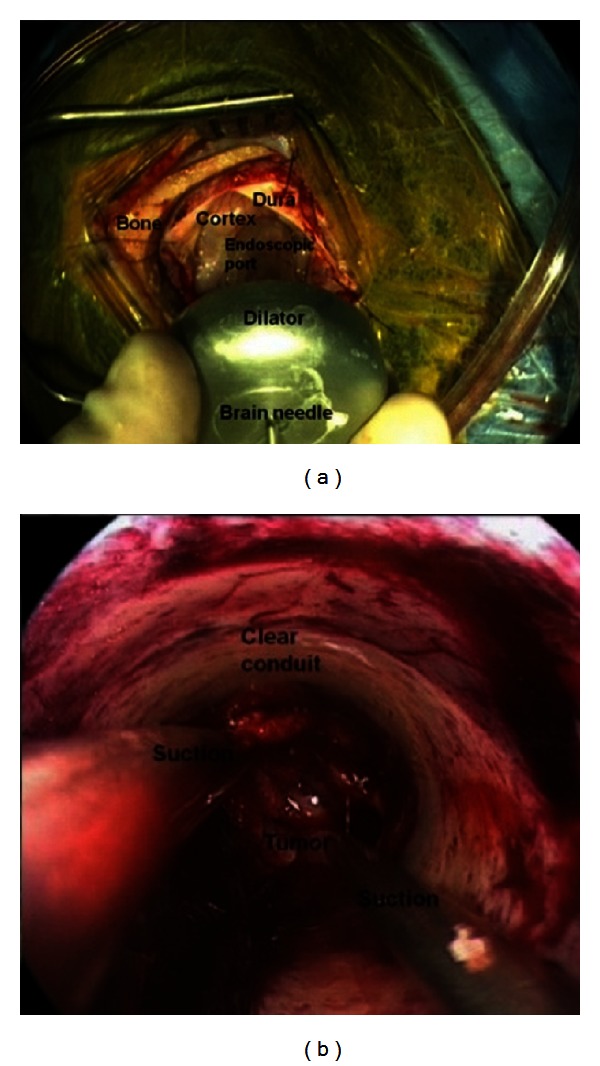
(a) View of the endoscopic port with dilator insertion and (b) surgical view through the endoscopic port.

The surgical technique of the authors is generally to use a twilight-awake-twilight model of anesthesia for these cases. The patient is fully off sedation for the entirety of the intradural portion of the case during which time the patient undergoes cortical mapping followed by continuous interaction with the neurophysiology team including reading flashcards, performing recall exercises, and answering simple questions. In this particular case, a temporal craniotomy was performed in the standard fashion centered over the middle temporal gyrus. Frameless image guidance was used to guide the placement of the bone flap, which was approximately 3 cm in diameter. Cortical mapping of the exposed temporal lobe did not reveal any changes in speech functions. The tumor was cannulated through the middle temporal gyrus using a previously described method [8] (Figure 2). Resection through the endoscopic port was relatively uneventful, except for two instances of fluency changes that occurred during tumor resection. Both were easily reversed by adjustment of the port trajectory. An aggressive resection was achieved. Pathological examination was consistent with glioblastoma multiforme (GBM). 

Postoperative MRI scan demonstrated a 93.3% volumetric resection of the tumor, with minimal residual tumor noted in the anteroinferior margin of the tumor bed (Figure 1). Postoperative recovery was uneventful, with the patient being discharged from the hospital on day 3.

At a one week postoperative followup, the patient was observed to have slight horizontal nystagmus that was noted postoperatively. He reported that his vision seemed to be improving. The patient did slur his speech on occasion; however, this was consistent with his baseline speech preoperatively. No new impairments of language or cognition were noted secondary to the surgery. The patient was undergoing speech and physical therapy and had been referred for radiation therapy and chemotherapy due to his GBM diagnosis. 

After 7 months, the patient had an MRI showing multifocal enhancement within the dominant hemisphere. MR spectroscopy was suggestive of multifocal tumor progression. The patient was subsequently treated with two cycles of avastin. At the 9-month followup, the patient has been noted to have improved T2 and FLAIR images consistent with the avastin effect. On the neurological exam, the patient was mildly dysarthric as had been noted prior to surgical intervention. The patient continued to demonstrate mild abnormalities with word finding but could recall 3/3 words in 5 minutes when given categorical clues. The patient had no other new motor, sensory, or cognitive defects noted on exam.

## 3. Discussion

Removal of brain tumors within eloquent brain regions can result in iatrogenic motor deficits, speech disorders, and complex cognitive deficits [2, 3]. However, for some tumors, including high grade gliomas, the extent of tumor removal is highly correlated with patient survival, pushing the surgeon to remove as much tumor as possible [2]. Awake craniotomy with cortical mapping can facilitate maximal tumor removal and help avoid iatrogenic injury in such cases [2, 12]. 

Numerous publications have described successful awake craniotomy protocols [13, 14] and retrospective results of successful use [2]. A recent prospective study examined 79 cases of awake craniotomy for supratentorial tumors, citing a 75% recovery from deficits versus an 8.9% worsening of symptoms postoperatively with 89.9% patient satisfaction and 1.3% mortality [15]. 

Of the factors portending good functional outcome in the removal of high grade gliomas in the temporal lobe using awake craniotomy, the most significant has been reported to be the distance of the surgical margin from the nearest language site [2]. This highlights the importance of minimizing collateral damage in surgical procedures in these critical areas. For deep-seated tumors, mapping of neurologic function can be more difficult than cortical mapping, and white matter dissection can be more extensive. EPS has been previously reported to minimize corticectomy volume and white matter dissection for supratentorial [8, 10] tumors, as well as intraventricular [9] and infratentorial [11] lesions. However, it should be noted that there is a lack of well-powered, controlled trials to investigate whether these new microsurgical techniques portend better outcomes. This paper represents the first known combination of awake craniotomy with EPS in a single operation. For selected cases in eloquent regions of the brain, the combination of these techniques may facilitate safe resection. 

## 4. Conclusion

The authors describe a case of awake craniotomy with cortical mapping followed by resection of an intraparenchymal tumor using an endoscopic port. In rare cases, the combination of these two techniques may be an option to facilitate deep tumor resection within eloquent regions of the brain.
